# Solitary fibrous tumor of the kidney: Magnetic resonance imaging characteristics in 4 patients

**DOI:** 10.1097/MD.0000000000011911

**Published:** 2018-08-24

**Authors:** Zongyu Xie, Guanghui Zhu, Liuquan Cheng, Jinhong Liu, Huiyi Ye, Haiyi Wang

**Affiliations:** a Department of Radiology, First Affiliated Hospital of Bengbu Medical College, Bengbu, Anhui; b Department of Radiology; c Department of Pathology, Chinese PLA General Hospital, Beijing, China.

**Keywords:** kidney, MRI, neoplasm, solitary fibrous tumor, spindle cells

## Abstract

To investigate the characteristics of magnetic resonance image (MRI) in solitary fibrous tumor (SFT) of the kidney.

MRI findings and clinical features of SFT of the kidney in 4 patients (2 men and 2 women with a mean age of 37.8 ± 8.7 years) were reviewed retrospectively. All patients were scanned by a 3.0-T magnetic resonance (MR) imaging system and the lesions were detected with emphasis on size, shape, location, margin, presence of pseudocapsule, signal intensity, degree of MR enhancement, and apparent diffusion coefficient (ADC).

The 5 tumors from 4 patients were located in renal parenchyma (n = 1), renal pelvis (n = 3), and renal capsule (n = 1). On MRIs, the tumors were round (n = 1) or oval-shaped (n = 4), and presented pseudocapsule (n = 2) and well-circumscribed margins (n = 3) can be found. On T2-weighted images (T2WIs), solid components of the tumor presented homogeneously mild hypointensity or isointensity (n = 4) compared with the renal cortex. On diffusion-weighted images (DWIs), the lesions showed normal or mild hyperintensity (n = 4) with mean ADC of 1.687 × 10^−3^ mm^2^/s. On dynamic contrast-enhanced MRIs, all lesions showed progressively mild enhancement. In the follow-up of 24 to 36 months after the surgery, 3 patients survived and 1 deceased.

The SFT of the kidney appeared as a circle or oval and presented homogeneously mild hypointensity or isointensity on T2WIs, hyperintensity on DWIs, and progressively mild enhancement on DCE MRIs.

## 1 Introduction

Solitary fibrous tumors (SFT) are the neoplasms of mesenchyma^[1]^ (or uncommon spindle cell neoplasms), which may occur in any part of the human body but mostly appear in the pleura.^[2]^ In a literature review, it is reported that SFT can be found in the head and neck regions,^[3,4]^ sinonasal cavity,^[5]^ orbit,^[6,7]^ extracranial region,^[8]^ larynx,^[9]^ parapharyngeal,^[10]^ pleura,^[11]^ buccal cavity,^[12]^ thigh,^[13]^ and paranasal sinuses.^[14]^ SFT of the kidney is a rare case but usually displays favorable prognosis.^[15]^ Only 55 cases of kidney SFT have been reported worldwide to date and most of them concentrated in the tumor size, location, and immunohistochemistry without description on the features of magnetic resonance images (MRIs) and follow-up situation.

Magnetic resonance (MR) imaging, based on its excellent soft tissue contrast, has been widely applied to detect and characterize diseases. Besides, a literature review on the adoption of MR scanner has reported its effectiveness on examining the SFT in different parts of the human body such as in the left thigh, head, and neck regions.^[16]^

Since inadequate cases reported the disease, SFT of kidney remains a medical mystery, leading to more difficulties in its diagnosis and treatment. Furthermore, images on SFT of kidney were also rare. In light of this, MRI now shoulders the responsibility to figure out the questions upon SFT of the kidney. Therefore, this article aims to investigate the MRI characteristics of SFT of the kidney, based on the cases from 4 patients.

## 2 Materials and methods

### 2.1 Patient characteristics

Four patients with pathologically confirmed soft fibrous tumor of the kidney were enrolled in this study from October 2013 to October 2015. Of these 4 participants, 2 were males and 2 were females with a mean age of 37.8 ± 8.7 years (range, 27–47 years). All patients received MRI examination on the tumor with emphasis on size, location, margin, signal intensity, and apparent diffusion coefficient (ADC). Three patients underwent DCE-MR imaging with enhanced hyperintensity, except the last one who was not suitable due to the large tumors in both kidneys.

This study was approved by the ethics committee of Chinese PLA General Hospital and the informed consent was obtained from each patient.

### 2.2 MRI examination

MRI examinations were performed by a 3.0-T MR imaging system (Signa; GE Medical Systems, Milwaukee, WI) with a surface phased-array coil (gradient magnet strength of 23 mT/m). Patients were scanned in supine position. Morphologic evaluation of the tumors was performed with following MR sequences: T1-weighted dual-echo image (T1WI) (repetition time [TR] = 260 milliseconds; echo time [TE] = 2.2–2.5 milliseconds, 5.5–5.8 milliseconds; FOV = 36–44 cm; matrix 256 × 192; section thickness 5 mm; gap 1 mm), T2-weighted image (T2WI) (TR = infinite; TE = 90–105 milliseconds; FOV = 36–44 cm; matrix 320 × 224; section thickness 5 mm; gap 1 mm), and 3-dimensional fat-saturated T1-weighted dynamic contrast-enhanced (DCE) image (TR = 3.0–3.9 milliseconds; TE = 1.2–1.6 milliseconds; FOV = 34–40 cm; matrix 288 × 224; section thickness 5 mm; interpolated section thickness −2.5 mm). Gadobenate dimeglumine (Magnevist; Schering, Berlin, Germany) was intravenously injected with a dose of 0.1 mmol (per kilogram of body weight) at a flow rate of 2 mL/s by a power injector (Spectris Solaris; Medrad, Indianola, PA), followed by a 20-mL flush of normal saline. DCE-MRI was performed in transverse plane at arterial phase, venous phase, and delayed phase. Diffusion-weighted imaging (DWI) was performed before DCE-MRI with b values of 0.80 s/mm^2^.

### 2.3 Pathologic analysis

All specimens were retrospectively examined by 2 pathologists with an experience in urine pathology for more than 10 years, neither of whom was blinded to the MRI findings.

## 3 Results

**Figure 1:**
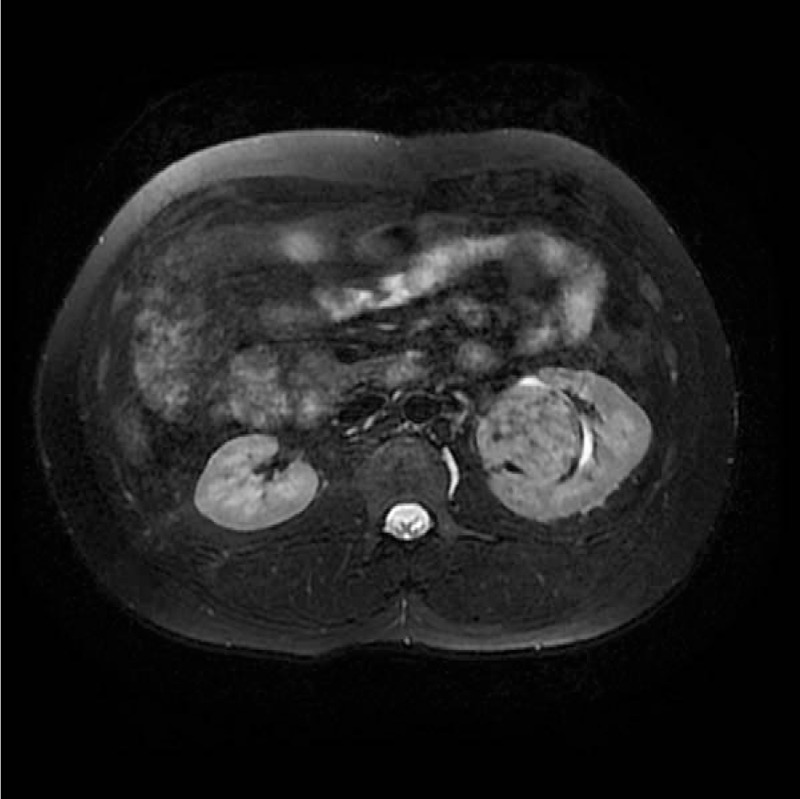
Image from a 27-year-old man with solitary fibrous tumor. An oval-shaped lesion located at the left kidney was observed with slight hyperintensity compared with the renal cortex.

**Figure 2:**
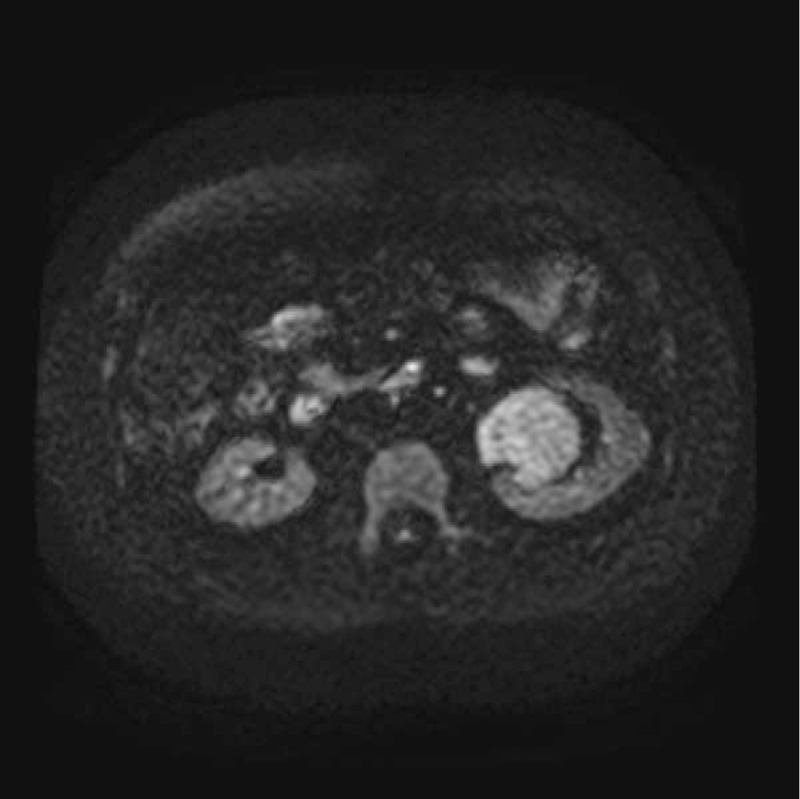
Example of a lesion demonstrates hyperintensity on dynamic weighted image (b value of 800 s/mm^2^).

**Figure 3:**
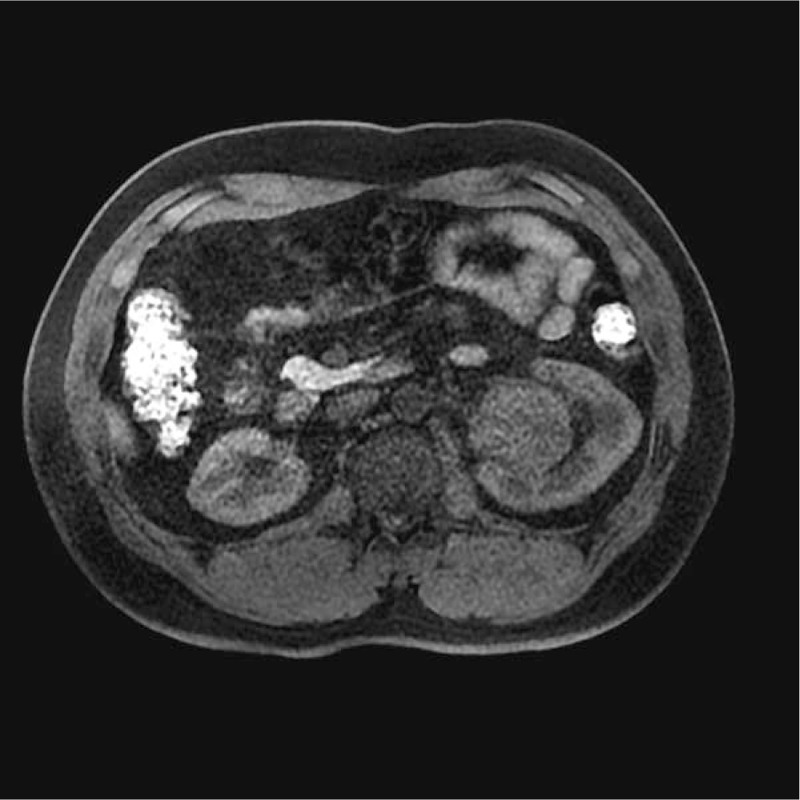
On precontrast T1-weighted dual-echo image, the lesion showed isointensity compared with the renal cortex.

**Figure 4:**
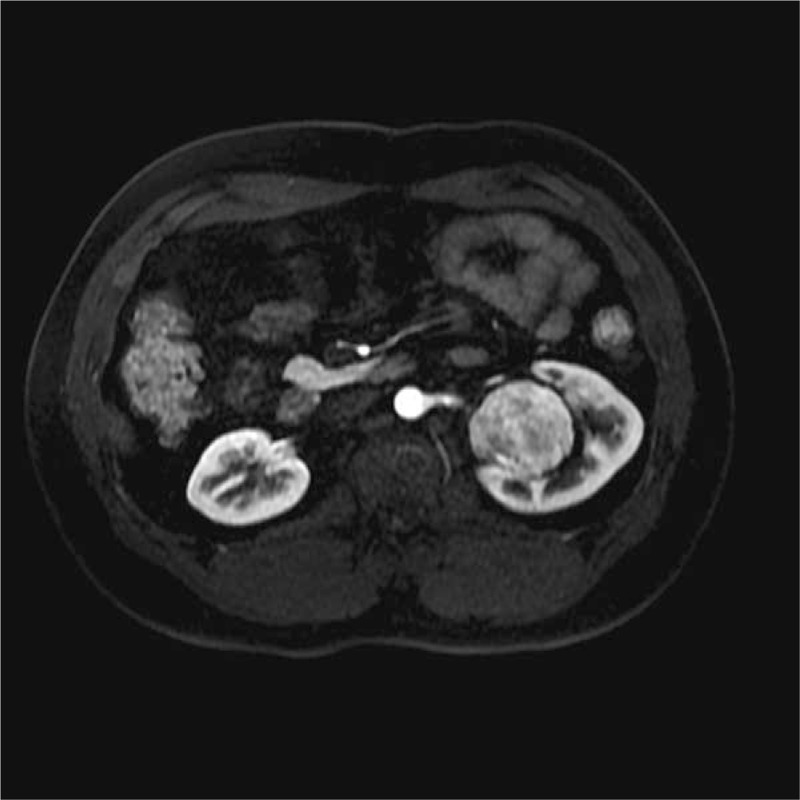
On dynamic contrast-enhanced magnetic resonance image, the arterial phase image showed unevenly mild enhancement.

**Figure 5:**
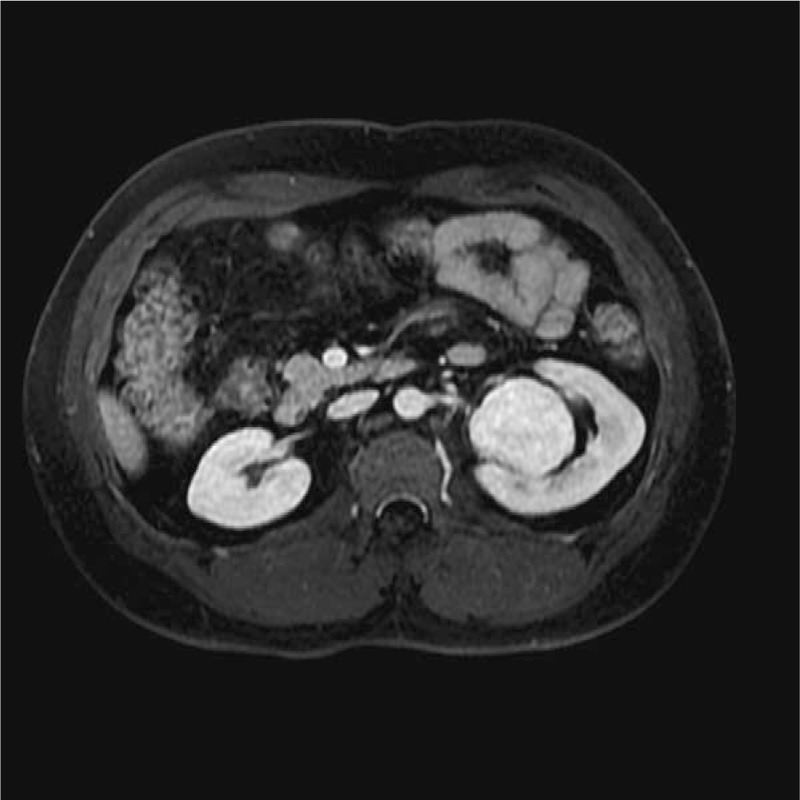
On dynamic contrast-enhanced magnetic resonance image, the venous phase image showed uniformly high enhancement.

**Figure 6:**
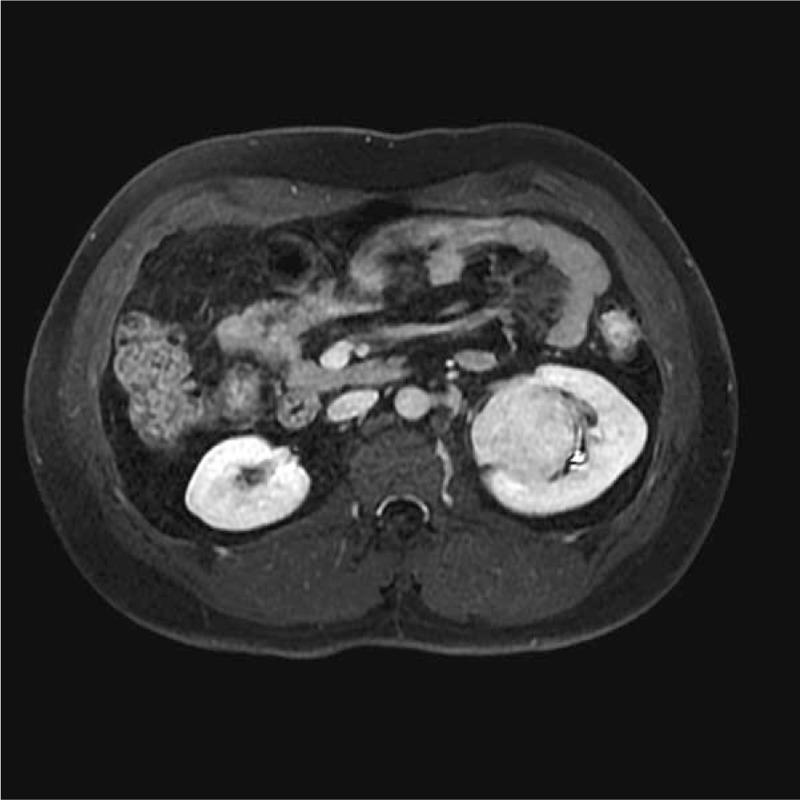
On dynamic contrast-enhanced magnetic resonance image, the delay phase image showed consistent enhancement.

The SFTs of the kidney were located in the renal parenchyma (n = 1), renal pelvis (n = 3), and renal capsule (n = 1). On the MR images, the tumors presented a round shape (n = 1) or an ovoid shape (n = 4). Well-circumscribed margins (n = 3) and pseudocapsule (n = 2) can be detected. On DWI findings, the lesions showed hyperintensity or mild hyperintensity (n = 5) (Fig. 1). The sizes of tumors on renal pelvis from the 3 survived patients were 4.5 × 3.5 × 2.8, 4 × 4 × 3.8, and 4 × 3.9 × 3.7 cm, respectively; and the sizes of 2 tumors on renal parenchyma and renal capsule from the deceased patient was 11.4 × 10.2 × 11.9 and 10.1 × 7.6 × 12.1 cm. The ADC of the 5 lesions were 1.56 × 10^−3^, 1.69 × 10^−3^, 1.58 × 10^−3^, 1.63 × 10^−3^, and 1.97 × 10^−3^ mm^2^/s, respectively, with a mean ADC of 1.687 × 10^−3^ mm^2^/s. Solid components of the tumors presented homogeneously mild hypointensity or isointensity (n = 4) compared with the renal cortex (Fig. 2) on T2WIs as well as homogeneous hyperintensity (n = 5) compared with the renal cortex (Fig. 3) on T1WIs. On DCE MR images (DCE-MRIs) (Fig. 4), all lesions presented progressively mild enhancement, which were confirmed by venous phase images (Fig. 5) and delay phase images (Fig. 6). In the follow-up of 24 to 36 months after the surgery, 3 patients survived and 1 deceased.

**Figure 7:**
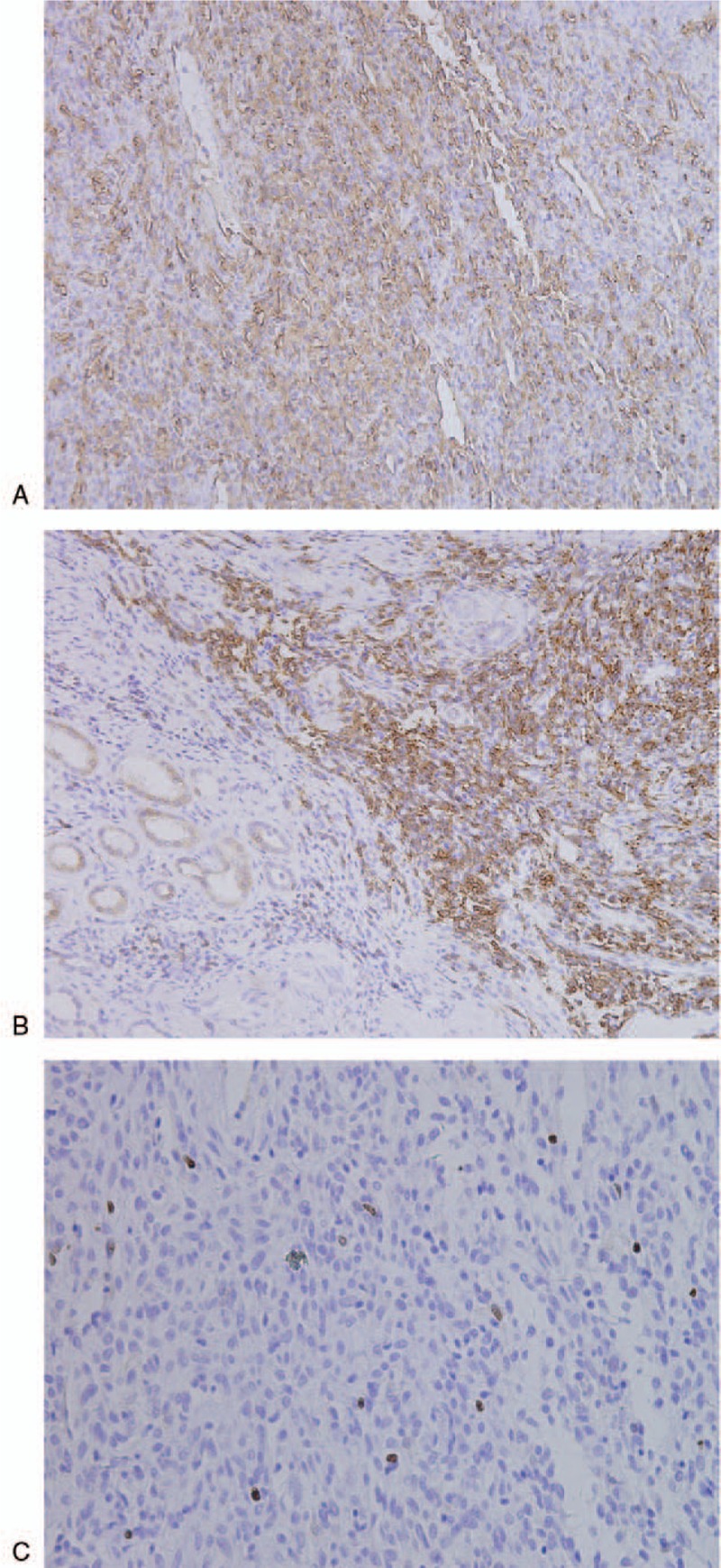
Immunostaining of tumorous cells deliver positive results. (A) Immunostaining of tumorous cells deliver positive results for CD34; (B) immunostaining of tumorous cells deliver positive results for bcl-2; (C) and immunostaining of tumorous cells deliver negative results for Ki-67.

Three patients underwent transurethral resection of the SFTs, and 1 patient with 2 tumors underwent posterior laparoscopic renal tumorectomy. Pathology examination revealed that the tumors were composed of short spindle cells and appeared with rich vessels, visible coarse collagen fibers, and branching veins. Immunochemical studies showed focal positivity for CD34 (Fig. 7A) and Bcl-2 (Fig. 7B), as well as weak focal positivity for Ki-67 (Fig. 7C).

## 4 Discussion

As a rare entity, SFT is a spindle cell neoplasm of mesenchymal, which may arise in any part of the human body but uncommon in the kidney.^[17]^ SFTs in the kidney have similar morphologic and immunologic features and biologic behaviors as the SFTs found elsewhere.^[18]^ The disease usually displays favorable prognosis and extremely infrequent retroperitoneal recurrence. Histologically 91% of the SFTs were benign and only 9% were malignant, which presented aggressive behavior.^[19]^ SFT of the kidney can demonstrate an aggressive character, while histopathological features and clinical results are still unknown. Furthermore, more difficulties are found in diagnosing SFT of the kidney, which may lead to unnecessary treatment without obvious malignant findings by current medical facilities.

Among the in hand cases, MRI examination on SFT^[3–14]^ showed high signal on T1WIs with undetermined characteristics. On T2WIs, the focal region presented high or other signals (for the lower part of the pleura signal), and DCE-MRIs revealed homogeneous enhancement features. Besides, fewer reports on SFT by MR scanner, mostly 1 or 2 cases, may lead to inaccurate conclusions. Among the 5 renal masses we examined, T1WIs of 2 lesions presented high or other signals, and 3 lesions showed low signal, which suggested unclear pattern. All the T2WIs showed low or isointense signal on SFT of the kidney. In some literatures, T2WIs also exhibited low or other signals in chest, which are consistent with our findings but different to other parts of the human body.

Despite the fact that MRI can expose the tumor from different perspectives thus reduces the prevalence of misdiagnosis, the technique has been scarcely applied to SFT of the kidney in particular. To the best of our knowledge, only 1 case from the National Center for Biotechnology Information reported the application of MRI on examining the SFT in the kidney, in which diffuse hypointense was revealed on T2WIs without central necrosis despite the large size of the renal mass.^[20]^ In our research, the MR images show that the SFTs were located at renal parenchyma, renal pelvis, or renal capsule, and the tumors are round shape or ovoid shape. On T2WIs, the solid components of the 4 tumors demonstrated homogeneously mild hypointensity or isointensity relative to the renal cortex. On DWIs, the 4 lesions showed normal or mild hyperintensity with mean ADC of 1.687 × 10^−3^ mm^2^/s.

In an MRI review,^[18]^ patients with kidney SFT basically treated by nephrectomy were followed up between 6 months to 4 years, and as a result most of these patients were healthy without recurrence. Only 2 cases observed metastasis: one is a 50-year-old man with cancer metastasis and recurrence 3 years after kidney removal. Another is a 48-year-old man with cancer metastasis after kidney removal for 8 years. In our study, the follow-up time for 4 patients was between 18 and 30 months. Three patients with only 1 lesion became healthy, and the last 1 with 2 large tumors lost contact, indicating that the patient may depart. Conventional MRI found that the 2 tumors in both kidneys were so large (11.4 × 10.2 × 11.9 and 10.1 × 7.6 × 12.1 cm) that this patient was not suitable for kidney surgery. As time goes by, the renal function gradually degenerated, thus indirectly resulting in fatigue and death finally. It was the first case that both the kidneys have such large SFTs at the same time.

To sum up, we found that the SFT of the kidney appears with the shape of round or oval; homogeneously mild hypointensity or isointensity on the focal masses was detected on T2WIs, hyperintensity or mild hyperintensity was observed on DWIs, and all lesions presented progressively mild enhancement on DCE-MRIs. Considering the results from our study and previous literatures, patient with kidney removal should regularly receive MRI to early address any possibility of relapse, which may contribute to death. Besides, the lack of research in the application of this promising technique on detecting SFT of the kidney request further studies for verification.

## Author contributions

**Conceptualization:** Guanghui Zhu.

**Data curation:** Haiyi Wang.

**Formal analysis:** Liuquan Cheng, Jinhong Liu.

**Investigation:** Liuquan Cheng, Jinhong Liu.

**Methodology:** Zongyu Xie.

**Project administration:** Guanghui Zhu.

**Software:** Jinhong Liu.

**Supervision:** Zongyu Xie.

**Validation:** Haiyi Wang.

**Writing – original draft:** Huiyi Ye.

**Writing – review & editing:** Zongyu Xie, Huiyi Ye.

## References

[1] Fursevich D Derrick E O’Dell MC Solitary fibrous tumor of the kidney: a case report and literature review. Cureus 2016;8:e490. 27014524 10.7759/cureus.490 PMC4792638

[2] Abeygunasekera AM Ginige AP Liyanage IS A solitary fibrous tumor of the kidney. J Cancer Res Ther 2015;11:662. 10.4103/0973-1482.138128 26458681

[3] Liu Y Li K Shi H Solitary fibrous tumours in the extracranial head and neck region: correlation of CT and MR features with pathologic findings. Radiol Med 2014;119:910–9. 24862631 10.1007/s11547-014-0409-9

[4] Kim HJ Lee HK Seo JJ MR imaging of solitary fibrous tumors in the head and neck. Korean J Radiol 2005;6:136–42. 16145288 10.3348/kjr.2005.6.3.136 PMC2685036

[5] Yang BT Song ZL Wang YZ Solitary fibrous tumor of the sinonasal cavity: CT and MR imaging findings. AJNR Am J Neuroradiol 2013;34:1248–51. 23413243 10.3174/ajnr.A3485 PMC7964567

[6] Zhang Z Shi J Guo J Value of MR imaging in differentiation between solitary fibrous tumor and schwannoma in the orbit. AJNR Am J Neuroradiol 2013;34:1067–71. 23306015 10.3174/ajnr.A3340 PMC7964636

[7] Kim HJ Kim HJ Kim YD Solitary fibrous tumor of the orbit: CT and MR imaging findings. AJNR Am J Neuroradiol 2008;29:857–62. 18272558 10.3174/ajnr.A0961 PMC8128581

[8] Motoori K Hanazawa T Yamakami I Intra- and extracranial solitary fibrous tumor of the trigeminal nerve: CT and MR imaging appearance. AJNR Am J Neuroradiol 2010;31:280–1. 19762459 10.3174/ajnr.A1702 PMC7964129

[9] Chang SK Yoon DY Choi CS CT, MR, and angiography findings of a solitary fibrous tumor of the larynx: a case report. Korean J Radiol 2008;9:568–71. 19039276 10.3348/kjr.2008.9.6.568 PMC2627233

[10] Jeong AK Lee HK Kim SY Solitary fibrous tumor of the parapharyngeal space: MR imaging findings. AJNR Am J Neuroradiol 2002;23:473–5. 11901021 PMC7975303

[11] Tateishi U Nishihara H Morikawa T Solitary fibrous tumor of the pleura: MR appearance and enhancement pattern. J Comput Assist Tomogr 2002;26:174–9. 11884769 10.1097/00004728-200203000-00002

[12] Shin JH Sung IY Suh JH Solitary fibrous tumor in the buccal space: MR findings with pathologic correlation. AJNR Am J Neuroradiol 2001;22:1890–2. 11733322 PMC7973830

[13] Riss O Mamlouk O Troufleau P CT and MR imaging of a solitary fibrous tumor of the thigh. J Radiol 2000;81:1643–6. 11104981

[14] Kim TA Brunberg JA Pearson JP Solitary fibrous tumor of the paranasal sinuses: CT and MR appearance. AJNR Am J Neuroradiol 1996;17:1767–72. 8896635 PMC8338285

[15] Usuba W Sasaki H Yoshie H Solitary fibrous tumor of the kidney developing local recurrence. Case Rep Urol 2016;2016:2426874. 27239363 10.1155/2016/2426874 PMC4864535

[16] Cheung F Talanki VR Liu J Metachronous malignant solitary fibrous tumor of kidney: case report and review of literature. Urol Case Rep 2016;4:45–7. 26793578 10.1016/j.eucr.2015.09.004 PMC4719796

[17] Kuroda N Ohe C Sakaida N Solitary fibrous tumor of the kidney with focus on clinical and pathobiological aspects. Int J Clin Exp Pathol 2014;7:2737–42. 25031693 PMC4097243

[18] Khater N Khauli R Shahait M Solitary fibrous tumors of the kidneys: presentation, evaluation, and treatment. Urol Int 2013;91:373–83. 24008397 10.1159/000354394

[19] Sfoungaristos S Papatheodorou M Kavouras A Solitary fibrous tumor of the kidney with massive retroperitoneal recurrence. A case presentation. Prague Med Rep 2012;113:246–50. 10.14712/23362936.2015.23 22980566

[20] Johnson TR Pedrosa I Goldsmith J Magnetic resonance imaging findings in solitary fibrous tumor of the kidney. J Comput Assist Tomogr 2005;29:481–3. 16012305 10.1097/01.rct.0000166637.24037.41

